# Fabrication and *In Vitro* Characterization of a Tissue Engineered PCL-PLLA Heart Valve

**DOI:** 10.1038/s41598-018-26452-y

**Published:** 2018-05-29

**Authors:** Anwarul Hasan, Sherif Soliman, Fatima El Hajj, Yuan-Tsan Tseng, Huseyin C. Yalcin, Hany Elsayed Marei

**Affiliations:** 1 Department of Mechanical and Industrial Engineering, College of Engineering, Qatar University, Doha, Qatar; 2 Biostage, Inc., Holliston, MA 01746 USA; 3 Biomedical Engineering, Faculty of Engineering and Architecture, American University of Beirut, Beirut, 11-0236 Lebanon; 4 Division of Qatar Cardiovascular Research Center, Sidra Medicine, Doha, Qatar; 5 Imperial College, NHLI, Heart Science Centre, Harefield, Middlesex, UB9 6JH United Kingdom; 6 Biomedical Research Center, Qatar University, Doha, PO Box 2713 Qatar

## Abstract

Heart valve diseases are among the leading causes of cardiac failure around the globe. Nearly 90,000 heart valve replacements occur in the USA annually. Currently, available options for heart valve replacement include bioprosthetic and mechanical valves, both of which have severe limitations. Bioprosthetic valves can last for only 10–20 years while patients with mechanical valves always require blood-thinning medications throughout the remainder of the patient’s life. Tissue engineering has emerged as a promising solution for the development of a viable, biocompatible and durable heart valve; however, a human implantable tissue engineered heart valve is yet to be achieved. In this study, a tri-leaflet heart valve structure is developed using electrospun polycaprolactone (PCL) and poly L-lactic acid (PLLA) scaffolds, and a set of *in vitro* testing protocol has been developed for routine manufacturing of tissue engineered heart valves. Stress-strain curves were obtained for mechanical characterization of different valves. The performances of the developed valves were hemodynamically tested using a pulse duplicator, and an echocardiography machine. Results confirmed the superiority of the PCL-PLLA heart valve compared to pure PCL or pure PLLA. The developed *in vitro* test protocol involving pulse duplicator and echocardiography tests have enormous potential for routine application in tissue engineering of heart valves.

## Introduction

Heart valve diseases are among the growing public health concern worldwide. About 25,000 deaths in the US and 3% of sudden deaths in the European Union occur annually because of cardiac valve defects^1,2^. These numbers are expected to triple in the next 50 years due to the increasing aging population^3^. Currently, biological valves made from human allografts or animal xenografts and mechanical valves made of metallic materials are used in surgical replacement of diseased/dysfunctional valves. The advantage of biological valves is their lower thrombotic risk compared to mechanical ones^4,5^, however, they are prone to the accumulation of calcium and lipids on the valve surface^6^ upon implantation. Besides, autologous tissue grafts that are used as a primary source for heart valve implants are in short supply^7^ while both allografts and xenografts such as those from porcine heart valves or bovine pericardium can last for only up to 10 to 15 years.

To increase the longevity of the implanted heart valve, the Ross procedure is applied whereby a diseased aortic heart valve is replaced with the native pulmonary heart valve of the same patient while the pulmonary valve is replaced with the implanted biological heart valve graft. The issue of poor mechanical properties of the implanted biological tissue grafts remain as a major limitation. Mechanical valves are made of strong durable materials with enhanced durability (20–30 years)^8,9^. However, they also have severe limitations, such as, high shear stresses of the blood flow on the mechanical valves result in platelet activation which results in a higher risk for thrombosis on the valve surface and embolism^10^. Therefore, patients with mechanical heart valves require blood thinners throughout the rest of their life. Besides, both the bioprosthetic and mechanical valves are unable to grow with time, due to which repeated surgeries might be required especially in young patients who might need multiple valve replacement operations over their lifetime. Recently tissue engineering has emerged as a promising solution for growing engineered viable tissues by incorporating living cells in suitable biocompatible and biodegradable scaffold materials^11,12^. The tissue engineered heart valves mimic native valve’s *in vivo* biological and physiological functions, and eventually become integrated with the patient’s native tissue^13,14^. The scaffolds usually have native extracellular matrix-like microstructure such as interconnected pores to promote cell migration inside the structure and support tissue structure^15–17^. They aim to match native mechanical properties such as stiffness in deformation to maintain its structure and function from the moment of implantation^18–21^. They should retain their anisotropy^22^, and should control the degradation rate while providing support for adequate tissue formation with none or minimal effect of toxicity and inflammation inside the body^23^. For that purpose, specific cell types are seeded into the scaffolds for an engineered tri-leaflet heart valve structure^24,25^. Recently a number of investigations have been performed on application of various biomaterials in development of tissue engineered heart valves^25–27^. Various biodegradable polymers such as Polycaprolactone (PCL), poly lactic acid (PLA), poly lactic-co-glycolic acid (PLGA) and poly glycolic acid (PGA) have been used in heart valve tissue applications^25^.

Van *et al*.^28^ applied PCL-fibrin composites coated with fibrin gel in heart valve tissue engineering. The applied PCL-fibrin composite was found to remain intact even after 10 million loading cycles *in vitro* and it showed good performance under simulated physiological flow conditions. Carbon nano fiber embedded PLGA polymer was also used in heart valve tissue engineering application^29^. In another study methacrylated gelatin (GelMA)-hydrogel was used in heart valve tissue engineering^30^. Masoumi *et al*.^31^ developed heart valve structure using PCL and poly glycerol sebasate (PGS) fibers with similar mechanical properties as the native heart valves.

In the present work, a tri-leaflet heart valve structure is developed using electrospun PCL -PLLA scaffold. Structural and morphological properties of the nano fiber scaffolds were investigated using scanning electron microscopy (SEM) and X-ray diffraction (XRD). The developed scaffold was assembled into a tri-leaflet heart valve shape, and seeded three dimensionally using porcine valvular interstitial cells under rotary dynamic seeding condition, followed by the surface seeding of C57/B1 mice cardiac stem cells (CSCs) on both sides. Spreading and metabolic activities of cells into the tri-leaflet heart valve structure was observed and Doppler echocardiography machine was used to assess the hemodynamics of the developed engineered tri-leaflet heart valve. Experimental results revealed that the resulting scaffold combines the stiffness and strength of PCL and the cell friendliness of PLLA together to form a foundation for engineered tri-leaflet heart valves. The *in vitro* cell culture studies demonstrated that the developed PCL-PLLA scaffolds were cytocompatible and showed substantial spreading and metabolic activities of seeded cells. The hemodynamic performance of the developed valve showed similar results as those obtained by using the commercial Edwards 2800 heart valve.

## Results and Discussion

**Figure 1:**
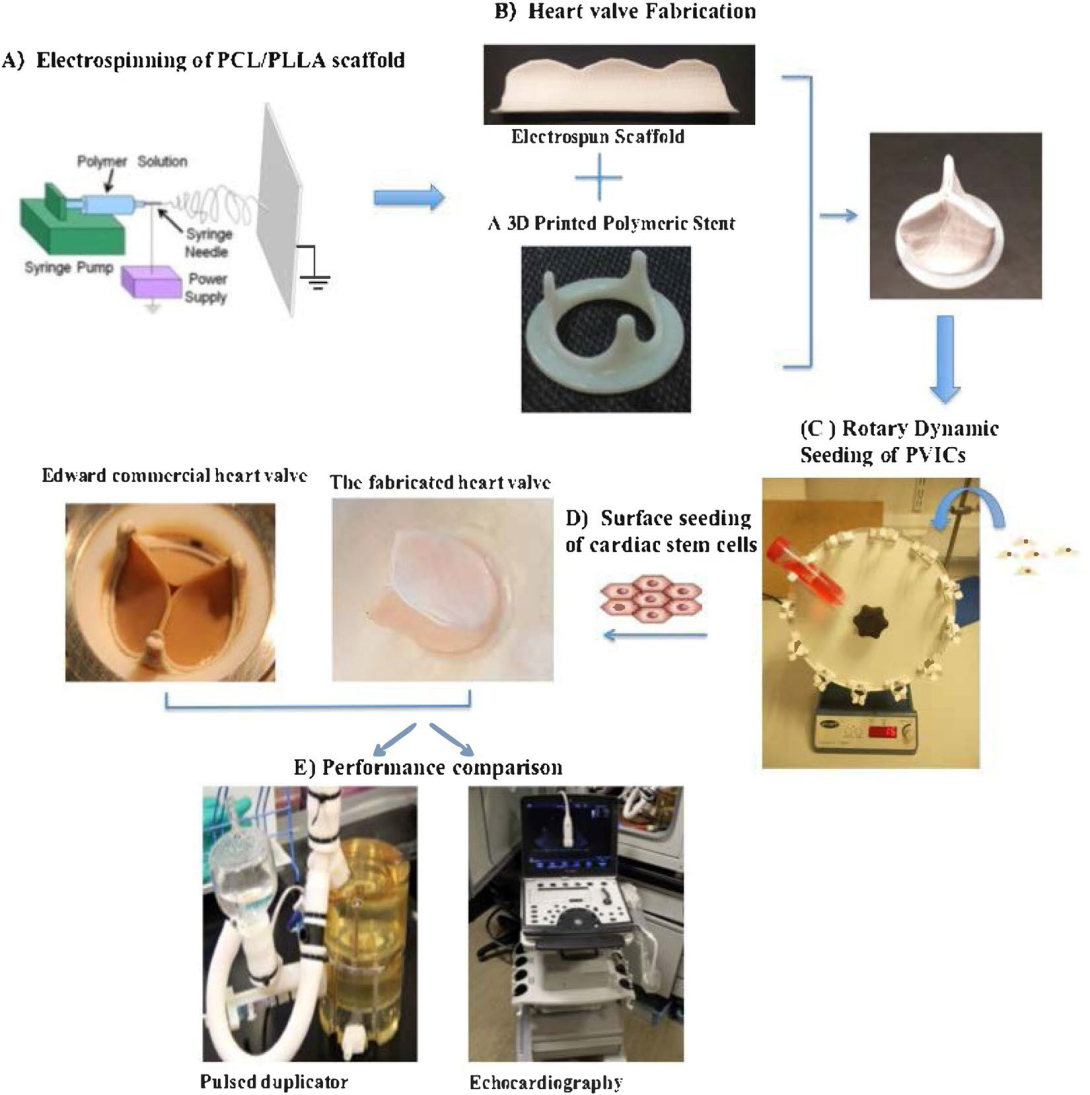
The fabrication process of the heart valve. (**A**) PCL: PLLA scaffolds were electrospun. (**B**) Heart valve leaflet shaped scaffold was wrapped around a printed stent. (**C**) Valvular interstitial cells were three dimensional-seeded using a rotary dynamic seeding, followed by (**D**) the surface seeding of cardiac stem cells on both sides. (**E**) The performance of the developed engineered heart valve structure was compared with an Edward commercial heart valve by studying the hemodynamics using a pulse duplicator system, and the Doppler flow velocity using an echocardiography system.

We developed electrospun PCL (M_n_ = 80,000), PLLA (M_n_ = 90,000), and PCL: PLLA blend, and mixtures nano fiber scaffolds of different ratios (Fig. 1A). At first, scaffolds were fabricated at different ratios of PCL and PLLA blending, namely, 100% PCL, 10:90, 30:70, 50:50, 70:30 and 90:30 ratios of PCL: PLLA, and 100% PLLA, all at an overall concentration of 10 wt.% as described in section 3.2. Based on the preliminary investigations of the mechanical properties and cell friendliness of the scaffolds, we selected a ratio of 30:70 PCL: PLLA as the optimum ratio for optimum combination of mechanical and cellular properties. The subsequent studies were performed using the PCL-PLLA samples of ratio 30:70 as mentioned in section 2. The structural, morphological, and mechanical properties of the scaffolds were characterized. The tri-leaflet heart valve structure was fabricated using 30:70 PCL: PLLA blend scaffold that combines the stiffness and strength of PCL and the cell adhesion affinity of PLLA. The developed scaffold was assembled into a tri-leaflet heart valve structure, Fig. 1B, which was three dimensionally seeded using porcine valvular interstitial cell under rotary dynamic condition (Fig. 1C), followed by the surface seeding of cardiac stem cells on both sides, (Fig. 1D). The rationale behind using a unique population of CSCs in this case was the fact that these cells have been reported to have the ability to generate endothelial cells *in vitro* as described in section 3.5.1. Substantial spreading and metabolic activities of cells into the tri-leaflet heart valve structure was observed. Qualitatively, the rotary seeding improved the cell colonization inside the scaffolds compared to static seeding. For hemodynamics evaluation, produced valves were exposed to physiological transient pressures in a pulse duplicator system (Fig. 1E). An echocardiography machine was used to measure orifice velocities, and results showed that the leaflets of PCL-PLLA valve could open and close effectively, similar to the commercial Edwards 2800 heart valve.

### Morphological analysis

**Figure 2:**
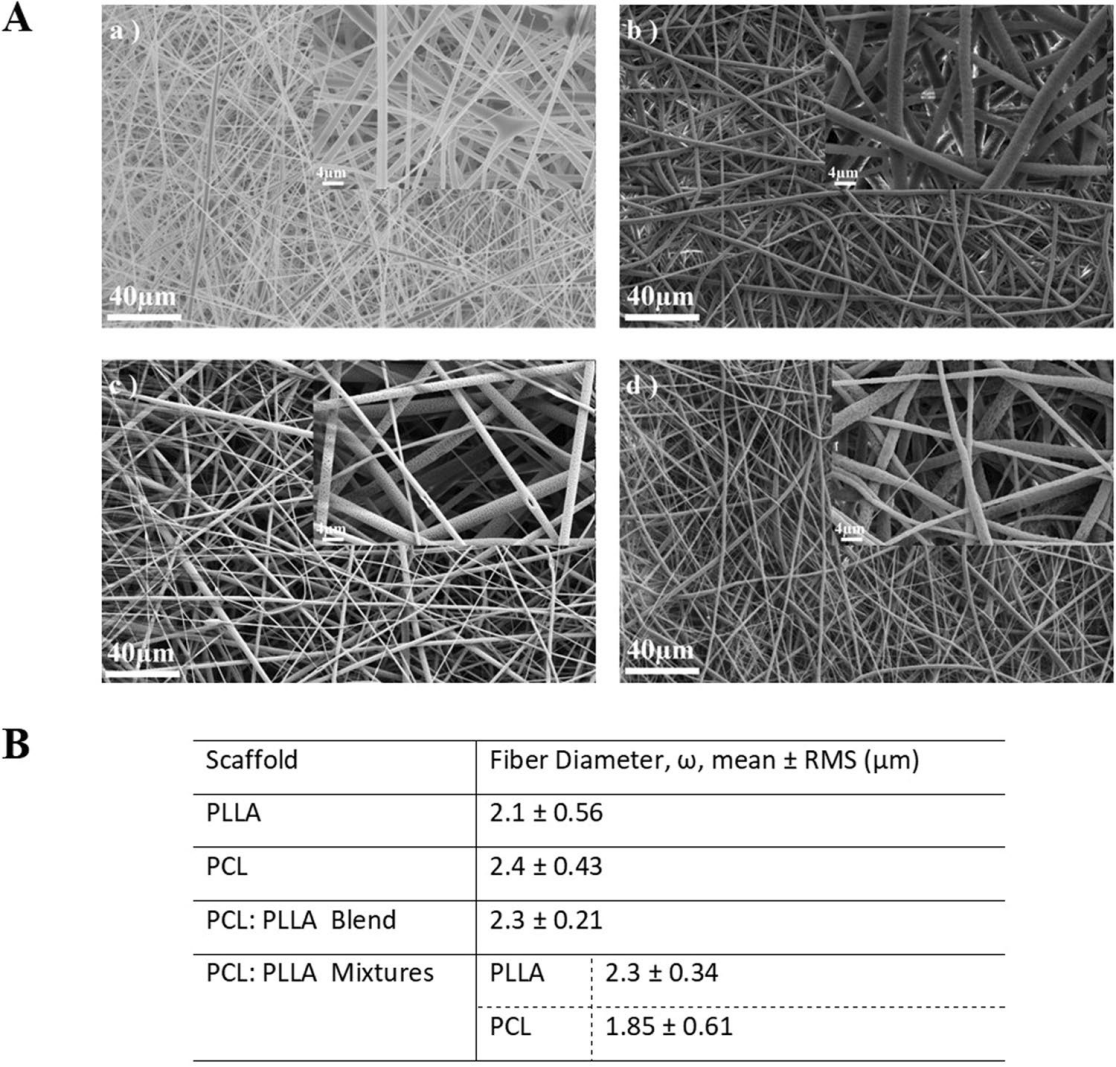
(**A**) Scanning electron micrographs of electrospun fibers, at concentration of 10% w/v, consisting of (a) PLLA (b) PCL, (c) 30:70 PCL: PLLA blend, (d) PCL: PLLA mixtures. (**B**) Mean and root mean square errors for the fiber diameter measurements.

The morphological analysis results of PCL, PLLA and the PCL: PLLA blend and mixture (at a ratio of 30:70) are shown in Fig. 2. The 30:70 PCL: PLLA co-mingled scaffolds showed a uniform distribution of both fibers and they were distinguishable through the SEM images (Fig. 2c,d). The final optimized scaffolds exhibited smooth fibers and relatively homogenous size in terms of diameter. The fiber diameters for all the groups showed values ranging between 1.85–2.4 µm, depending on the process settings. In the case of the fiber mixtures, the density of both fibers was characterized by counting the number of fibers that intersected a line drawn across the middle of the image. This check rendered similar percent coverage of both polymers when the target rotation rate was set at 60 rpm under the processing conditions.

### Crystallinity

**Figure 3:**
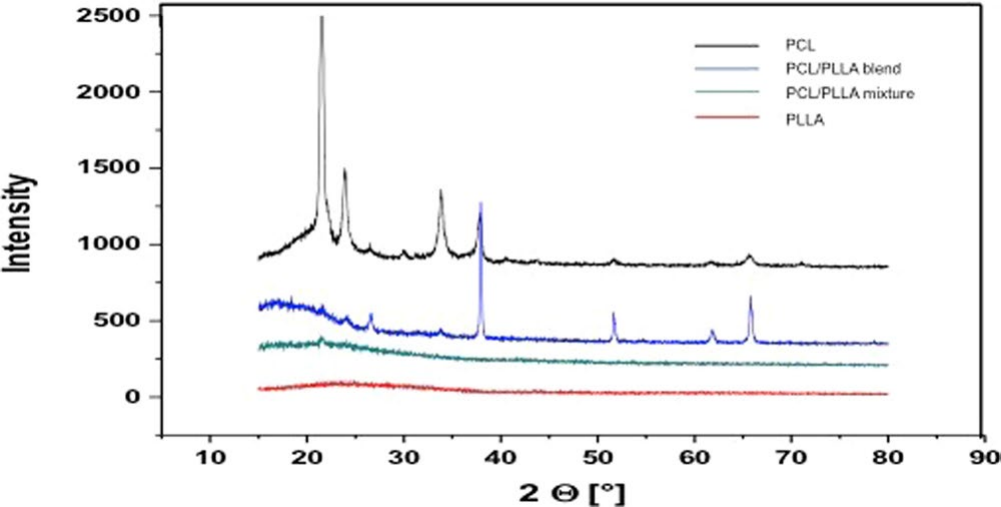
XRD spectra relative to PCL, PLLA, 30:70 PCL: PLLA blend, and 30:70 PCL: PLLA mixtures scaffolds. PCL showed sharp peak at 2θ of 22° and a relatively low intensity peak at 23.6°, showing the crystalline nature of PCL. The PLC/PLLA blend has shown a close crystalline structure to PCL but the most intense peak of the blend shifts from the one in the angle 22° to the one at 38, perhaps because a change on the orientation at the time of doing the blend. The PCL: PLLA mixtures were found to be semi-amorphous and more similar to pure PLLA, while PLLA fibers exhibit an amorphous material structure, which indicated that the crystallization of PLLA could be prevented by the processing technique.

XRD analysis was performed to characterize the structural properties of PCL, PLLA, 30:70 PCL: PLLA blend, and PCL: PLLA mixtures. XRD patterns (Fig. 3) showed sharp peak at 2*θ* of 22° and a relatively low intensity peak at 23.6° which shows the crystalline nature of PCL. The PCL and PCL: PLLA blend showed a close crystalline structure. It is obvious that in case of the blends, PCL enhanced the crystalline structure of the composite. The PCL: PLLA mixtures were found to be semi-amorphous and more similar to pure PLLA. PLLA fibers exhibited an amorphous material structure, which indicated that the crystallization of PLLA could be prevented by the processing technique^32^. PCL fibers showed a crystalline phase. The data highlights also a difference between the blends and the mixtures, showing that the latter ones are more similar to pure PLLA fibers. In fact, the amorphous behavior is predominant with very little evidence of the highest peak typical of PCL fibers at 2θ = 22°. Instead, the blends resemble purer than PCL, as we can see almost all the PCL peaks but also the presence of PLLA peaks, although of lesser relative intensity. Remarkably, the most intense peak of the blend shifted from the one in the angle 22° to the one at 38°, perhaps because of a change on the orientation at the time of doing the blend (Fig. 3).

### Mechanical Properties

**Figure 4:**
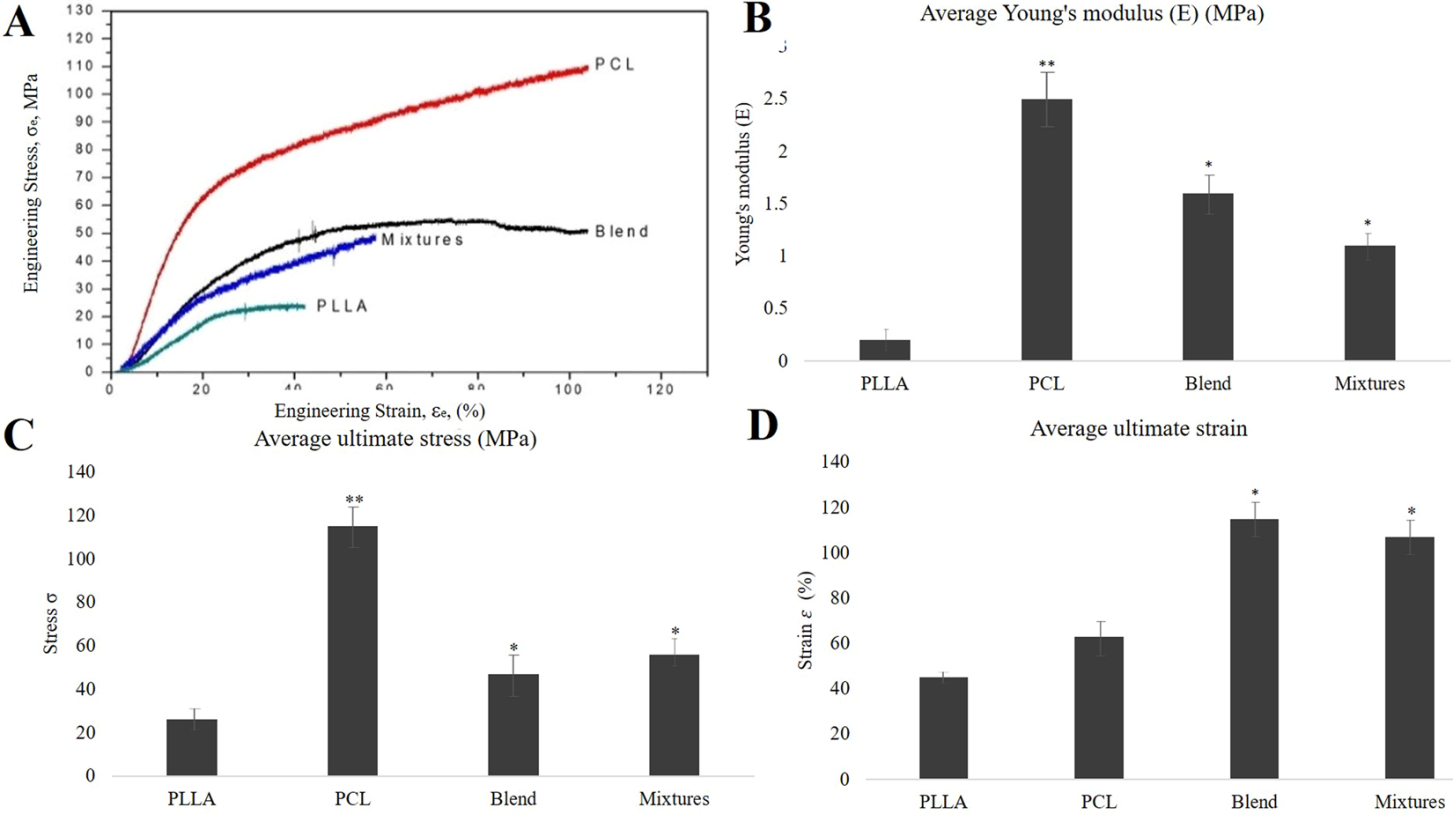
Mechanical properties of the different scaffolds. (**A**) Representative Stress-Strain curves for PCL, PLLA, and 30:70 PCL: PLLA blend and PCL: PLLA mixtures. A fixed crosshead rate of 10 mm/min was utilized in all cases, and the results were taken as an average of five tests. The bar diagrams represent the average Young’s modulus (**B**) average ultimate stress (**C**) and the average ultimate strain (**D**) of the different scaffolds. The Young’s modulus of the different scaffolds revealed that the stiffness decreased in the following order: PCL, PCL: PLLA blend, PCL: PLLA mixtures, and PLLA. Ultimate stresses are in comparable ranges with similar trends. The material ductility increased in the following range: PCL, Blends, Mixtures, PLLA, which can be taken as an indirect indication of higher material toughness. The softening behavior after the stress peak is different and relates to different failure modes. While PLLA behaves a multilayer composite that fails by delamination due to the propagation of a Mode II crack, the PCL and PCL: PLLA blends and their mixtures behaved similarly to fiber bundles failing by Mode I crack. Tukey HSD test has been performed for the groups with *p < 0.05, **p < 0.01 between its counterpart group for its significance.

The mechanical properties in terms of stress-strain curves, Young’s modulus of elasticity, the ultimate tensile stress and the ultimate tensile strain from two representative tests are shown in Fig. 4 for PCL, PLLA, and PCL: PLLA (30:70) blend, and mixture. While the amount of strain for different materials in the linear elastic ranges were comparable, PCL turned out to be the stiffest among all in terms of modulus of elasticity. The stiffness decreased in the following order, PCL->Blends->Mixtures->PLLA. The Young’s modulus appeared to be 2.5, 1.6, 1, and 0.2 MPa for PCL, blend, mixtures, and PLLA respectively. Ultimate stresses also varied in comparable ranges with similar trends. The material ductility increased in the following order PLLA->Blends->Mixtures->PCL, such as the average ultimate stress were 28, 46.6, 55.1, and 112 MPa respectively. The softening behavior after the stress peak were different and related to different failure modes. While PLLA behaved like a multilayer composite that fails by delamination due to the propagation of a Mode II crack, the PCL and PCL: PLLA blends and their mixtures behaved in similar way as fiber bundles failing by Mode I crack. Recently the mechanical properties of animals (bovine, ovine, and porcine), human and tissue engineered heart valve constructs from literature over several decades were summarized in an excellent review^19^ published by Hasan *et al*. The heart valves were found to be inherently highly anisotropic in nature, i.e. the elastic modulus and ultimate tensile stress of the valve leaflets were higher in the circumferential direction compared to those in the radial direction. A significant difference between human valves and those of common animals such as porcine, bovine, or ovine valves were evident. In addition, the animal heart valves were much weaker compared to human heart valves. The literature data showed that the elastic modulus, ultimate tensile stress and the strain at the ultimate tensile stress for human native heart valves were 15 MPa, 2.6 MPa and 22% in the circumferential direction and 2 MPa, 0.4 MPa and 30% in the radial direction respectively. These nano fibers scaffolds, in the current study showed these values to be in the range of 0.25 to 2.5 MPa, 20 to 110 MPa and 40 to 110% respectively. Thus, the tensile strength and the elongation of our developed scaffolds appear to be higher compared to those of native human heart valves. The elastic modulus of the scaffolds (0.25 to 2.5 MPa) were much lower compared to that of the native heart valves in in the circumferential direction (15 MPa), however they were higher compared to that of the native heart valves in the radial direction (2 MPa). Thus, developed scaffolds possess strong mechanical properties, and thereby have strong potential for application in heart valve replacements.

### Preparation of PCL: PLLA tri-leaflet heart valve structure

**Figure 5:**
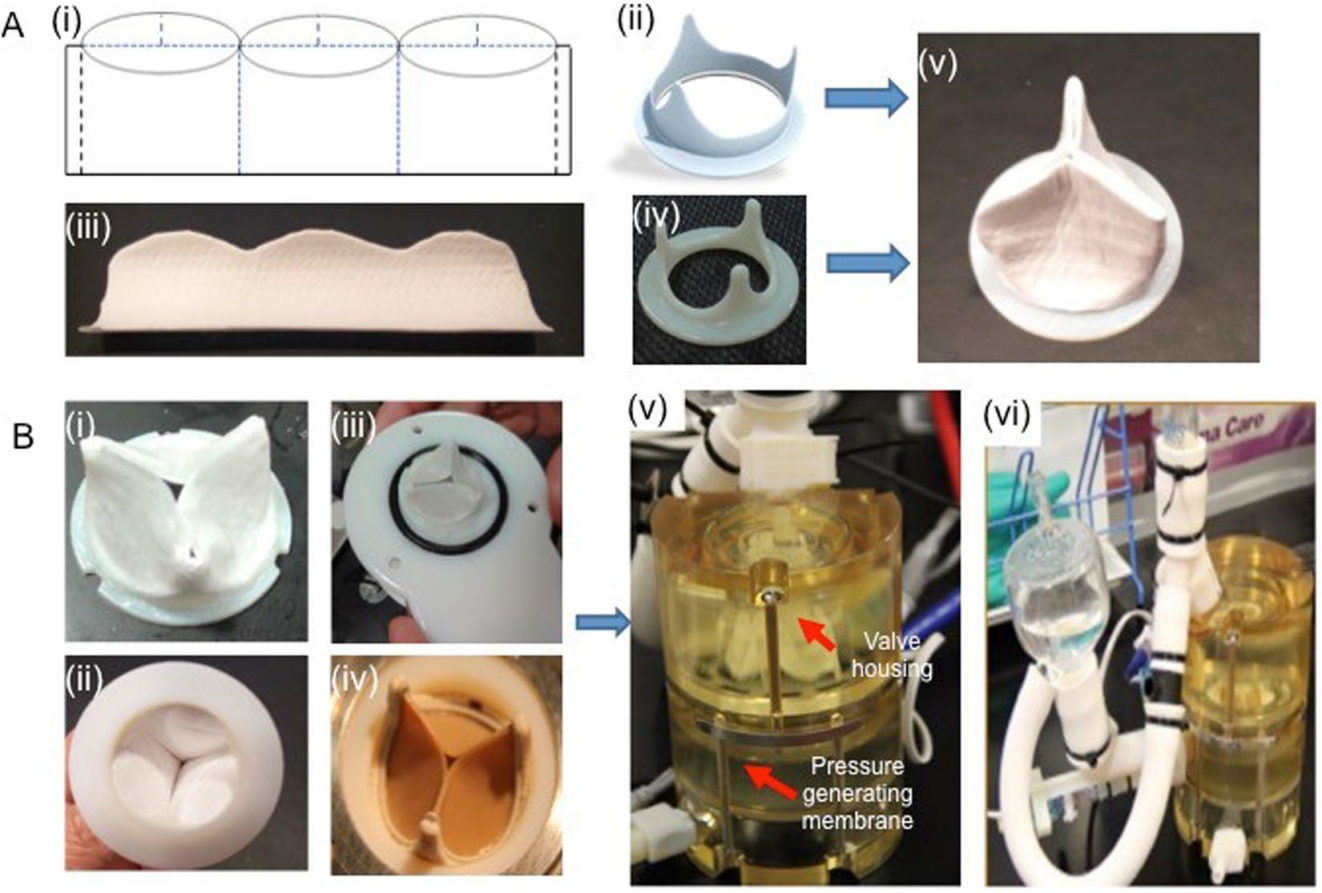
Preparation of PCL: PLLA heart valve leaflet shaped scaffold. (**A**) Fabrication of the heart valve leaflet. (i) A stent was designed using a CAD software (Solid works) to provide the supporting structure of the valve. (ii) Schematic of wrapping around the designed stent, then it was 3D printed using object 260 (Stratasys Ltd) with Vero white material. (iii) PCL: PLLA scaffold was cut into shape with a metal cutter. (iv) It was wrapped around the printed stent. Finally, (v) it was joined with the 3D printed stent using melted PCL. (**B**) Testing the fabricated PCL: PLLA valve using pulse duplicator system. (i) Top view, (ii) bottom view of the valve, (iii) the fabricated PCL: PLLA valve mounted on a disk for installing on pulse duplicator to be compared to (iv) commercial heart valve (Edwards 2800 valve). (v) The valve was housed into (vi) the pulse duplicator system. The pulse duplicator system used to test the fabricated valve subjected to artificial flood (25% glycerol and 0.9% saline solution) flow with pressure of 30 mmHg and heart rate 70 bpm in 10 h.

The 30:70 PCL: PLLA blend was chosen to fabricate the engineered tri-leaflet heart valve as shown in Fig. 5A, since it showed one of the best performance in the *in vitro* cell culture studies. Some earlier studies also showed that PCL: PLLA scaffolds exhibit better mechanical and biological behavior compared to PCL or PLLA alone^33^. PCL: PLLA blend showed a potential application in tissue engineering as a simple, effective, scalable alternative material^34^. The performance of our developed PCL: PLLA valve was tested using a pulse duplicator system and compared to commercial heart valve (Edwards 2800 valve) (Fig. 5B). It was subjected to artificial blood (25% glycerol and 0.9% saline solution) flow with a physiological pressure for 10 h.

### *In vitro* cell culture studies

**Figure 6:**
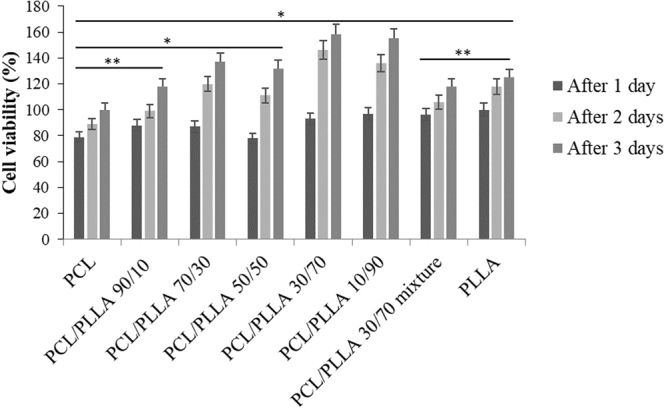
MTT assay was tested using cardiac stem cells (CSCs) after 1, 2 and 3 days in culture. CSCs exhibited strong metabolic activities on all of the scaffolds tested, with no evidence of toxic effects. In case of the blends and the mixtures, the results were better compared to those for pure PCL. In particular, the metabolic activities improved with the increase in the percentage of PLLA in the blends, from 0 to 90%. An evident boost in metabolic activities was exerted by 30:70 PCL: PLLA blend. Tukey HSD test has been performed for the groups with *p < 0.05, **p < 0.01 between its counterpart group for its significance.

**Figure 7:**
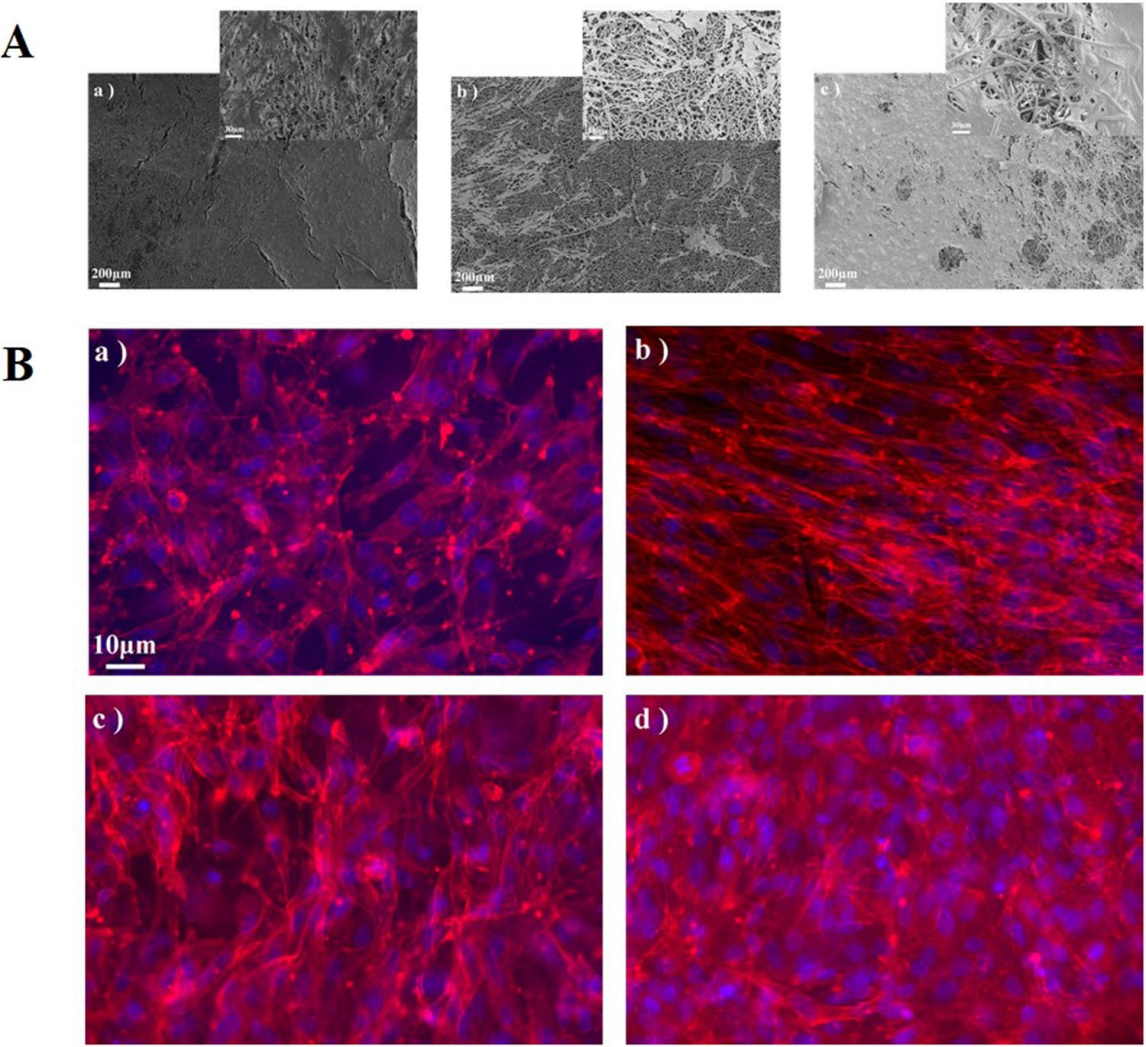
*In vitro* cell culture of cardiac stem cells (CSCs) seeded on the developed tri-leaflet heart valve structure. (**A**) Scanning electron micrographs of pre-seeded valve structure consisting of (a) PLLA, (b) PCL, and (c) 30:70 PCL: PLLA blend. The samples were sputter-coated with a thin layer of gold for 2 minutes, and their morphological structures were observed using SEM apparatus (FE-SEM, LEO SUPRA1250, Japan) at an accelerating voltage of 8 kV. (**B**) IF images of cardiac stem cells (CSCs) seeded on the valve structure developed by (a) PCL, (b) PLLA, (c) 30:70 PCL: PLLA blend, (d) 30:70 PCL: PLLA mixtures. The scale bar shown applies to all images. The CSCs were able to colonize the pores between fibers created from the deposition, and to spread to the fibers. The red color shows the actin fibers of CSCs while the blue color shows the cell nucleus.

The cell viability results of CSCs seeded on different scaffolds tested after 24, 48 and 72 hours are shown in Fig. 6. The MTT assay showed that CSCs exhibited sufficient metabolic activities in case of all of the scaffolds tested, with no evidence of toxic effects (Fig. 6). Our SEM results (Fig. 7A,B) show the spread of cells on the surface of scaffolds. The immunofluorescence (Fig. 7B) pointed that stem cells were able to colonize the pores between fibers created from the deposition. In case of the blends and the mixtures, the results were better in terms of metabolic activities as compared with pure PCL. In particular, the metabolic activities improved with the increase in the percentage of PLLA in the blends. A boost in metabolic activities is observed for 30:70 PCL: PLLA blend. Our results demonstrate the cytocompatibility of the materials employed, and a substantial spreading and metabolic activities of seeded cells onto the constructs.

### Cell colonization of porcine valvular interstitial cells (pVICs) and CSCs

**Figure 8:**
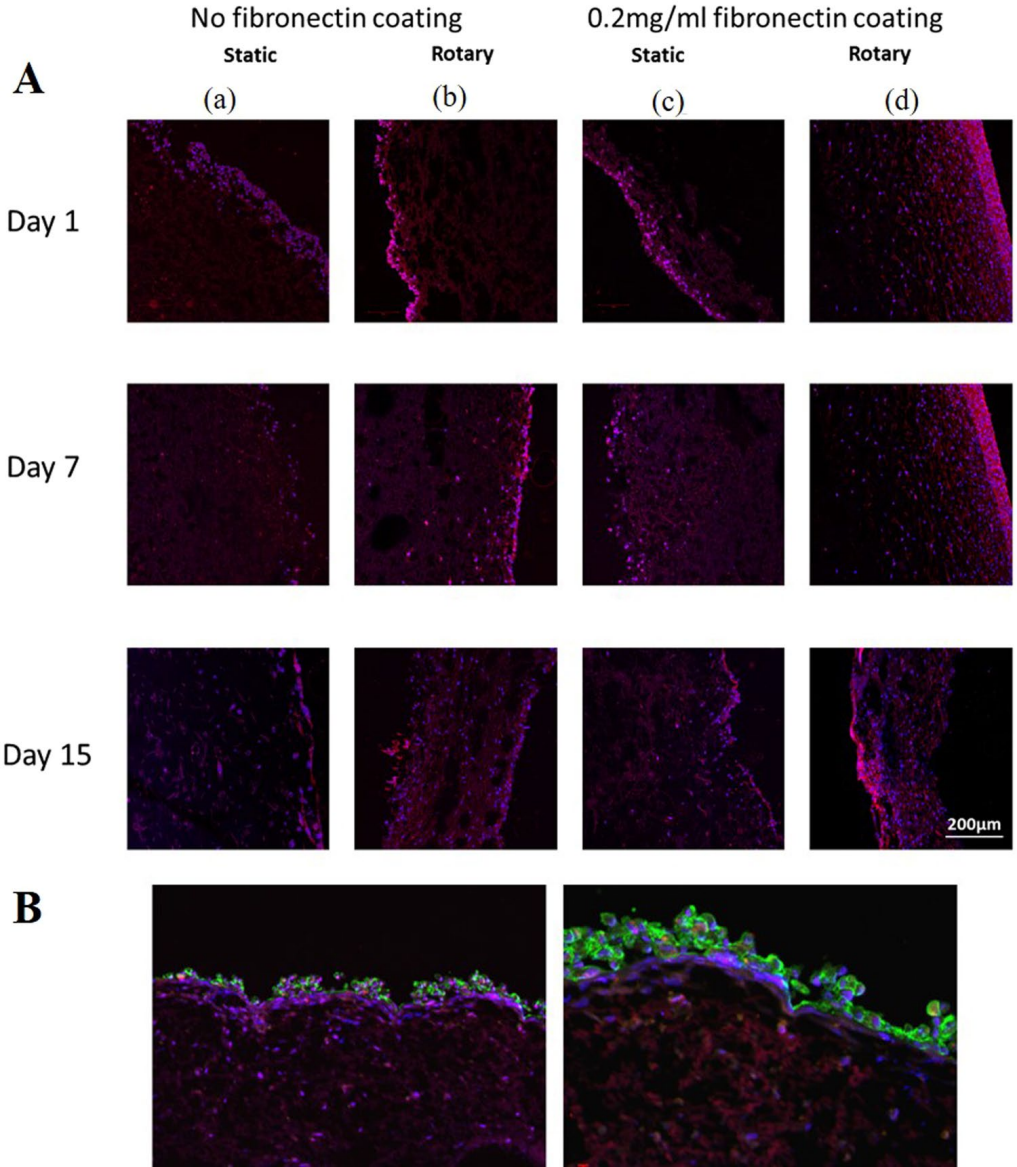
PCL: PLLA tri-leaflet heart valve structure seeded with CSCs and pVICs. (**A**) 3-dimensional seeding of pVICs in the scaffolds (a) static seeding without fibronectin coating, (b) dynamic seeding without fibronectin coating, (c) static seeding with 0.2 mg/ml fibronectin coating and (d) dynamic seeding with 0.2 mg/ml fibronectin coating. PVICs are dynamically seeded and stained with CF 90 Red in the middle. Cell nuclei were stained with DAPI blue. The results show that in dynamic seeding cells penetrated inside the scaffold better and exhibited improved cell colonization over a period of 15 days in comparison with static seeding for both fibronectin coated and uncoated valve structure. (**B**) A scaffold seeded with both pVICs and CSCs. The CSCs were stained using CellTracker^TM^ green CMFDA (ThermoFisher, with an excitation wavelength of 492 nm and emitting wavelength of 517 nm) to distinguish them from the pVICs. The CSCs tagged with cell tracker were seeded on the surface. The multilayer structure shows the pVICs inside the scaffold and CSCs (green) on the surface. The blue color shows the cell nucleus stained using DAPI.

The static and rotary seeding of pVICs in scaffolds under all four different seeding conditions are shown in Fig. 8A. And the multilayer valvular structure resulted from the three-dimensional seeding of pVICs followed by the surface seeding of CSCs are shown in Fig. 8B. Figure 8A shows that the rotatory seeding substantially improved the cell colonization of the tri-leaflet valve structure over the 15 days period in comparison with the static seeding for both fibronectin coated and uncoated scaffolds. The rotatory seeding enhances mass transport of nutrient and waste, thus allowing the complete cell colonization by 15 days. In addition, Fibronectin coated valve structure showed enhanced rate of cell colonization at 7 days period compared to the uncoated scaffold. The fibronectin provide the bioactive surface that can enhance cell adhesion to the valve structure; therefore enhance the rate of colonization. Figure 8A,B thus reveal that a combination of rotary seeding of pVICs and static surface seeding of stem cells (or the appropriate type of endothelial cells) can be an effective method for development of implantable tissue engineered heart valve structures.

### Performance study of the developed Engineered Heart Valve structure

#### Study of hemodynamics of the developed heart valve using a pulse duplicator

**Figure 9:**
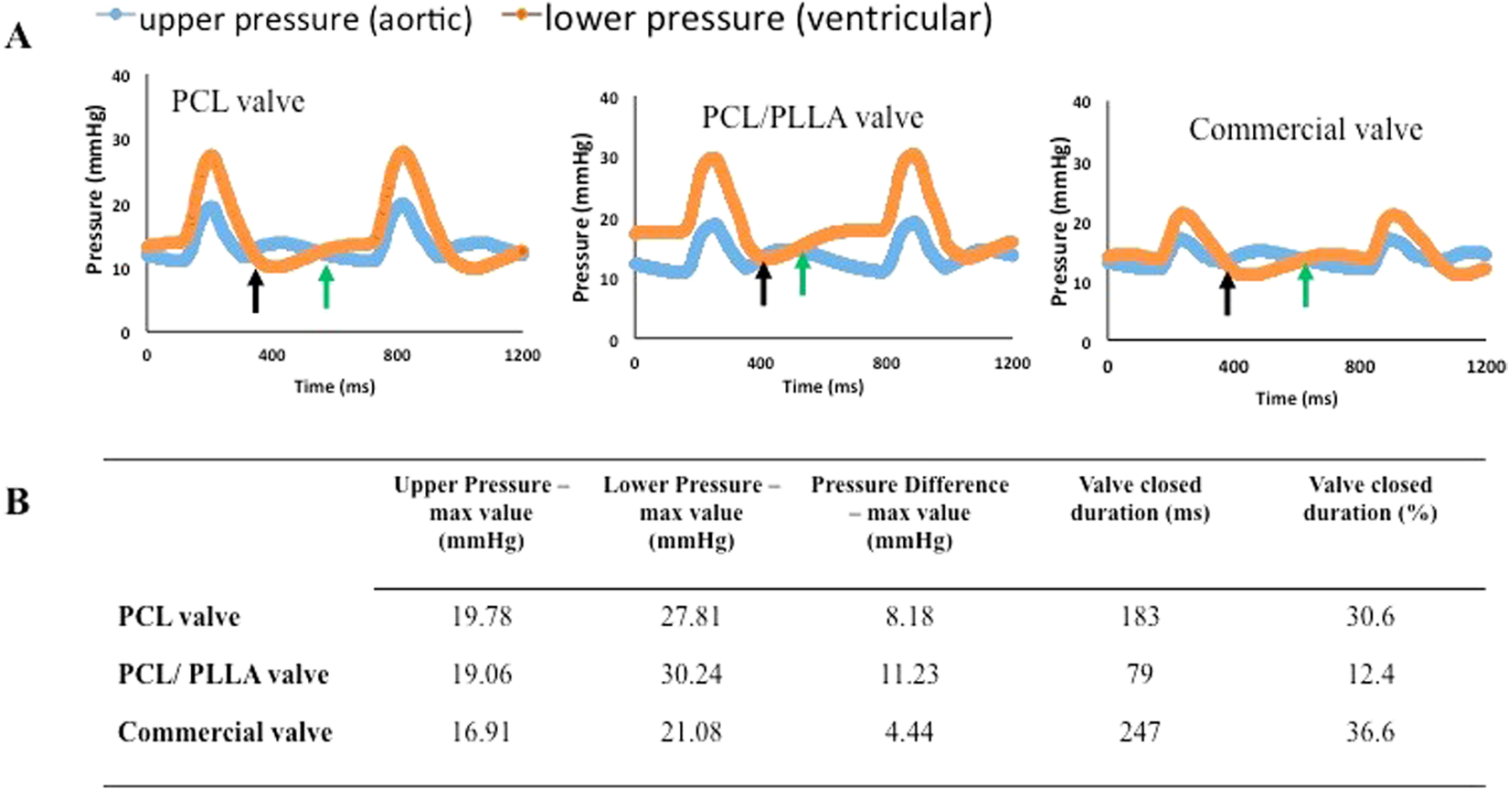
Pulsed duplicator system pressure readings for the tested valves. (**A**) Pressure readings for (i) PCL valve at concentration 10% w/v, (ii) PCL: PLLA valve, and (iii) commercial valve (Edwards 2800 valve). The first arrow (black) shows the time point where the lower pressure went below the upper pressure; at this point, the valve closes. The leaflets stay closed until the second arrow (green) at which point lower pressure becomes higher than upper pressure. The interval between these two arrows represents the closure duration of the valve. PCL valve stay closed more than the PCL: PLLA valve but less than the commercial valve. (**B**) Pressure readings such as the upper and lower pressure, pressure difference, and valve closed duration for the tested valves. Less pressure difference is generated in the PCL valve compared to PCL: PLLA valve. The pressure difference for PCL valve is still higher than the commercial valve.

Figure 9 shows the summary of results from pulse duplicator readings. Three valves were tested in the Aptus Pulse Duplicator System. Aptus control panel enables to define heart beat per minute (bpm) and systolic pressure (in mmHg). We selected 70 bpm heart rate and 20-mmHg systolic pressure for the tests. At this time, the Aptus unit in our laboratory can generate only pulmonary physiologic pressures (~20 mmHg). The system monitors pressures near the inlet and outlet of the valves. The ventricularis surface of the valve faces bottom of the bioreactor. Pressure near the inlet of the valve is named as lower pressure whereas pressure near the outlet of the valve is named as upper pressure. Figure 9A shows the changes of these two pressures in time for the three tested valves. The first arrow (black) shows the time point where the lower pressure went below the upper pressure value; at this point, the valve closes. The leaflets stay closed until the second arrow (green) at which point lower pressure becomes higher than upper pressure. The interval between these two arrows represents the closure duration of the valve. PCL valve stay closed more than the PCL: PLLA valve but less than the commercial valve. Figure 9B summarizes pressure value readings as well as time duration of closure for tested valves. According to these results, PCL valve generates 8.18-mmHg max pressure difference compared to PCL: PLLA valve with 11.23 mmHg and commercial valve with 4.44 mmHg. PCL valve is closed at 30.6% of the cardiac cycle whereas PCL: PLLA valve is closed at 12.4% and commercial valve at 36.6%. These results show that even though PCL valve functions better than the PCL: PLLA valve, both PCL and PCL: PLLA valves function close to the commercial valve.

#### Doppler echo cardiography of the developed engineered heart valve

**Figure 10:**
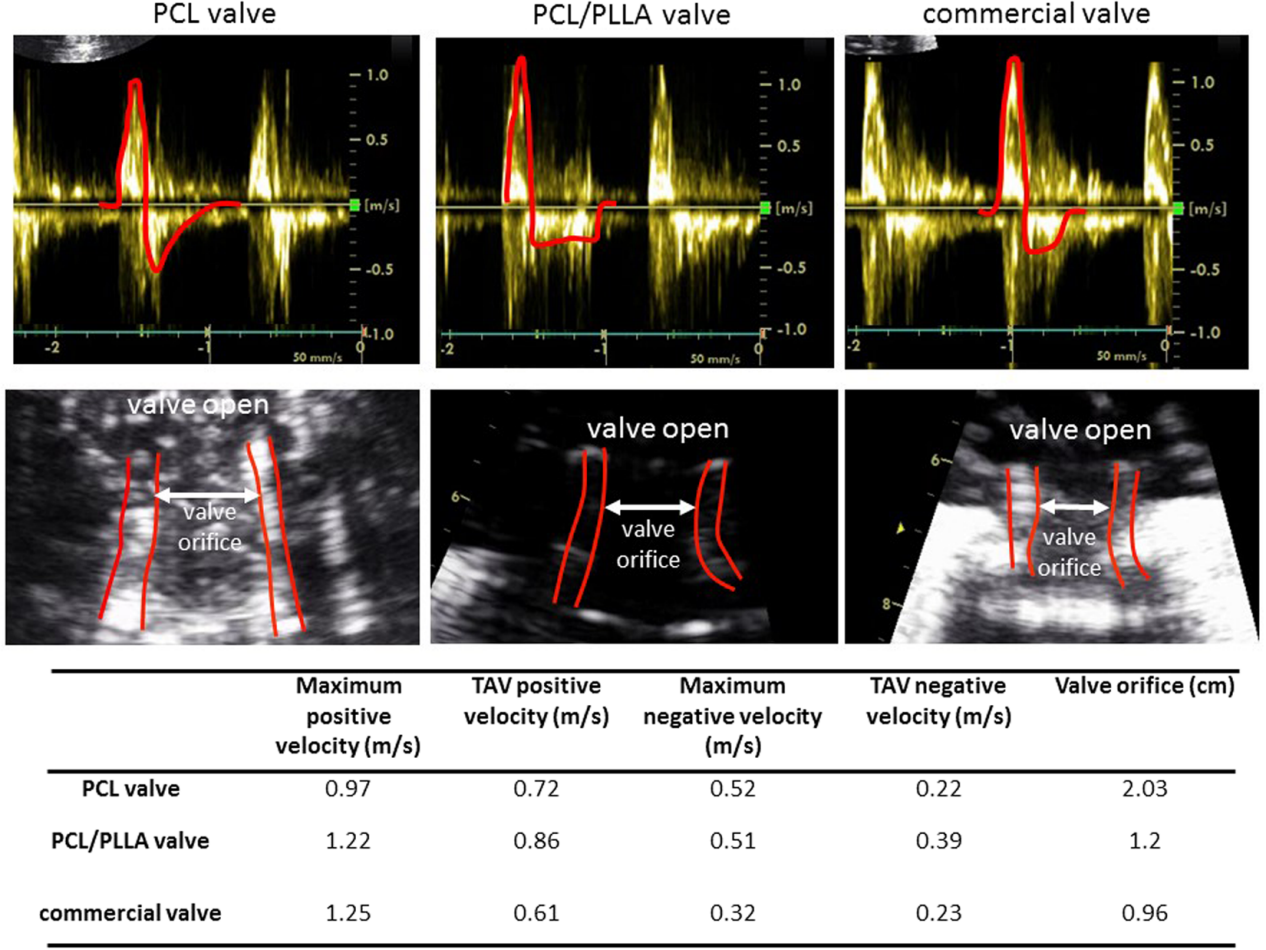
Doppler echo based experimental setup to test the fabricated heart valve compared to commercial valve. PCL valve results in maximum positive velocity value were lower than both the PCL: PLLA and the commercial valve, but time averaged value (TAV) of positive velocity for PCL valve was higher than the commercial valve and lower than the PCL: PLLA valve. Negative velocity values for PCL valve were lower than PCL: PLLA valve but higher than commercial valve showing regurgitation was more for the PCL valve compared to the commercial valve. PCL valve orifice size is more than both the commercial valve and the PCL: PLLA valve showing its superior opening characteristics compared to other two.

Figure 10 summarizes Doppler velocity measurements and b-mode orifice size measurements for the tested valves. According to these results, PCL: PLLA valve results in similar positive velocity value compared to commercial valve and higher positive velocity value compared to PCL. This result shows that expected wall shear stress levels would be similar for the PCL: PLLA valve and commercial valve. Time averaged value (TAV) of positive velocity for PCL valve is higher than the commercial valve and lower than the PCL: PLLA valve. Negative velocity values for PCL: PLLA valve are similar to PCL valve and these are higher than commercial valve suggesting regurgitation is more for the PCL: PLLA and PCL valves compared to the commercial valve. When we look at valve orifice sizes, PCL: PLLA valve orifice size is similar to the commercial valve and smaller than the PCL valve Similar to pulse duplicator pressure readings, these results show that PCL valve functions slightly better than the PCL: PLLA valve. Both PCL and PCL: PLLA valves function close to the commercial valve.

## Materials and Method

### Materials

In this work, we used PCL (molecular weight ~ 70,000–90,000) and PLLA (molecular weight ~ 80,000–100,000) polymers purchased from Sigma Aldrich to fabricate electrospun scaffolds. Dulbecco’s modified eagle medium (DMEM), Fetal bovine serum (FBS), non-essential amino acid in low glucose (1000 ng/ml), trypsin, paraformaldehyde, and phosphate buffer saline (PBS) solutions were purchased from Sigma Aldrich. Penicillin, streptomycin and glutamine were obtained from Gibco. Leibowitz medium was obtained from Worthington Biochemical Corporation, Lakewood, NY. Immunostaining kits such as those for the Actin-fiber staining and the cell nucleus were purchased from Invitrogen. The CellTracker™ Green CMFDA (5-chloromethylfluorescein diacetate) was purchased from ThermoFisher Scientific. All other materials were purchased from Sigma Aldrich.

### Preparation of electrospun PCL-PLLA scaffolds

PCL-PLLA nano-micro fiber scaffolds were fabricated at 10% weight concentration using electrospinning technique (Fig. 1A). At first, scaffolds were fabricated at different ratios of PCL and PLLA blending, namely, 100% PCL, 10:90, 30:70, 50:50, 70:30 and 90:30 ratios of PCL: PLLA, and 100% PLLA, all at an overall concentration of 10 wt.%. The scaffolds were electrospun under different process parameters in order to determine the optimal conditions for obtaining reproducible fibers with morphological properties that are similar to the control samples, as shown in Table 1. Based on the preliminary investigations of the mechanical properties and cell friendliness of the scaffolds of different PCL, PLLA ratios, we selected a ratio of 30:70 PCL: PLLA as the optimum ratio for optimum combination of mechanical and cellular properties. All subsequent investigations were performed using the PCL-PLLA samples of ratio 30:70, prepared in two different ways. One way was blending of PCL and PLLA solution before electrospinning, while the other method was to electrospun PCL and PLLA fibers simultaneously from two different syringes to form a PCL-PLLA mixed fiber mesh. In the electrospinning chamber, the humidity was kept at 30% and the temperature at 23 °C.

**Table 1:** Electrospinning processing conditions for the different electrospun scaffolds.

	Material	Solvent	Concentration (% w/v)	Screen Distance (cm)	Voltage (kV)	Flow Rate (mL/h)	Needle Gauge	Spinning Time (min)
	PLLA	DCM	10	20	20	6	21	30
	PCL	DCM	10	16.5	20	8	21	30
		DCM	10	20	15	6	22	30
		DCM	10	15	15;	6	21	30
		DCM	10	15	15	5	21	30

PCL, PLLA, their blend, and their mixtures (of ratios PCL: PLLA 30:70) were prepared in different polymer concentrations. Blend scaffolds made of PCL and PLLA were modulated by changing the polymer concentration. PCL and PLLA were electrospun simultaneously from two different syringes to form a mixed fiber mesh.

### Characterization of the electrospun scaffolds

#### Scanning Electron Microscopy

Four types of samples namely, 100% PCL 100% PLLA, 30%:70% PCL: PLLA blend and 30%:70% PCL: PLLA mixture scaffolds were selected for SEM and other characterizations. The samples were sputter-coated with a thin layer of gold for 2 minutes, and their morphological structures were observed using SEM apparatus (FE-SEM, LEO SUPRA1250, Japan) at an accelerating voltage of 8 kV.

#### X-Ray diffraction

The voltage used was 20 kV, TCD of 15 cm, and the scaffolds were scanned from 2θ = 15° to 2θ = 80° using Cu Kα radiation source at ambient temperature.

#### Mechanical testing

Uniaxial tensile mechanical test was performed on PCL, PLLA, and 30:70 PCL: PLLA blend and mixture scaffolds (n = 5) using a mechanical tester (Instron, model no. 5943). The stress-strain curves of the scaffolds were obtained from uniaxial tensile load-displacement graphs. Tensile test was carried out according to the ASTMD882 type V with applying crosshead speed of 10 mm/min. The Young’s modulus was calculated from the slope of the initial linear section of the stress-strain curve. The ultimate tensile stress was measured as the maximum stress before the failure while the ultimate strain was taken as the point of breakage for each sample.

### Fabrication of heart valve leaflet structure using PCL-PLLA scaffolds

A stent was designed using a computer aided design (CAD) software, Solid works, to provide the supporting structure of the valve. This was 3D printed using object 260 (Stratasys Ltd) with Vero white material. The scaffold was cut into shape with a metal cutter, followed by wrapping the scaffold around the 3D printed stent and fixing it on the stent using melted PCL as required to give it the shape of a tri-leaflet heart valve. The structure was then ready for cell seeding.

### *In vitro* cell culture

#### Cell isolation and cell culture

The porcine valve interstitial cells (pVICs) were isolated from freshly dissected porcine valves where whole hearts from 18 to 24-month-old pigs were obtained from an abattoir. Aortic valve cusps were removed within 12 hours of slaughter and pVICs were isolated through collagenase digestions as previously described^35^. More specifically, Valves were dissected from the root, washed several times with PBS, and further incubated in collagenase for 45 min at 37 °C. Collagenase was then deactivated by FBS, and cells were pelleted followed by re-suspension in DMEM. PVICs were seeded into 6- wells plates and expanded as required.

The CSCs were isolated from female C57/B1 mice (6 weeks old) using a protocol as described in forte *et al*.^36^. At first myocardial tissue was obtained from anesthetized mice, which was minced and incubated at 4 °C overnight with 0.05% trypsin in a solution of 0.02% EDTA in PBS. At first myocardial tissue was obtained from anesthetized mice, which was minced and incubated at 4 °C overnight with 0.05% trypsin in a solution of 0.02% EDTA in PBS. Trypsin inhibitor was used to stop the enzymatic reaction, and tissue fragments were incubated in collagenase II (1500 U) in Leibovitz medium. The fragments were centrifuged for 10 minutes at 800 g. Finally, the pellet was re-suspended in complete medium, filtered through a 70-μm cell strainer, and incubated at 37 °C in 5% CO_2_. The next day the medium and the non-adhering cells were removed and substituted with fresh medium, which was changed every other day to remove all cardiomyocytes. After 10–15 days, the non-myocytic cells (NMCs) were trypsinized and harvested. From this nonmyocytic fraction, a resident population of CSCs characterized by the expression of Sca-1 but lacking the expression of blood lineage markers and c-kit were obtained, expanded and cultured as described in^36^. Even though the heart valve surface is known to contain valvular endothelial cells, the rationale behind using the unique population of CSCs in this case was the fact that these cells have been reported to be self-renewing, clonogenic and multipotent, with the ability to generate endothelial cells *in vitro* (in the presence of 10% FBS and dexamethasone)^37^. Animal care and experimental procedures were performed according to the protocol and the guidelines approved by the institutional animal care committee in the Imperial College London, UK. Also all experimental methods were carried out in accordance with relevant guidelines and regulations.

#### MTT assay of CSCs

MTT assay of CSCs cultured on PCL, PCL: PLLA blends (90:10, 70:30, 50:50, 30:70, 10:90), PCL: PLLA mixtures 30:70, and 100% PLLA scaffolds was measured to study their metabolic activity after 1, 2, and 3 days. The MTT solution prepared in differentiation medium of concentration 0.45 mg/mL was added to each well for 4 h at 37 °C. Then, the scaffolds were placed in 1.5 mL polypropylene tubes, and 0.2 mL of DMSO was added. The constructs were broken apart using a tissue homogenizer to dissolve the formazan crystals formed by cells presenting mitochondrial metabolic activity. The absorbance was measured using ultra violate (UV) spectrophotometer at 540 nm. In addition, CSCs cultured on the PCL, PLLA, and 30:70 PCL: PLLA blend scaffolds were used for SEM analysis. The samples were fixed in 2% (v/v) aqueous glutaraldehyde and dehydrated in a graded series of water ethanol solutions for 10 min each and then critical point dried. Thin sections of the dried samples were gold sputtered and examined by SEM at 25 kV. Structural characterization of the CSCs cultured on PCL, PLLA, 30:70 PCL: PLLA blend, and 30:70 PCL: PLLA mixtures engineered valve were performed using immunofluorescence microscopy.

#### Immunofluorescence (IF) staining

For IF staining, the cells were fixed after a continuous monolayer of CSCs was formed. The cells were fixed in 4% paraformaldehyde for 30 min, and permealized using 0.1% Triton X for 20 min. 1% bovine serum albumin (BSA) in PBS was used to block nonspecific sites for 1 h. Finally, the cytoskeletal actin fibers of the cells were stained using Alexa Fluor 488 (red) and the nucleus of the cells were stained with DAPI (blue).

#### Static and Rotary seeding of the scaffold

PCL: PLLA tri-leaflet heart valve structures were sterilized in 70% ethanol for 15 mins on a roller mixer, followed by 3 × 15 minutes washes in sterilized PBS. The valve structure was coated with and without 0.2 mg/ml of human fibronectin for 15 mins before seeding. Two seeding methods for days 1, 7 and 15 were used for comparison. (i) Static seeding: the scaffold was placed in a 48 wells cell crown insert with seeding density of approximately 6000 cells/mm^3^. (ii) Rotatory seeding: the scaffold went through rotary seeding with pVICs at a density of approximately 6000 cells/mm^3^ by placing the scaffold in a 50 ml Tube Spin® Bioreactor (TPP) with 25 ml of cell suspension in the complete medium (Fig. 1C,D).

#### PVICs and CSCs co-culture

For co-culture of pVICs and CSCs, the CSCs were tagged with cell tracker^TM^ green CMFDA (ThermoFisher, with an excitation and emitting wavelengths of 492 nm and 517 nm respectively) using the protocol prescribed by the supplier. The tagging of CSCs with the cell tracker prior to cell seeding was performed so that they could be distinguished from the pVICs upon cell seeding. Four seeding conditions were followed: (i) the tri-leaflet engineered valve went through static seeding of pVICs followed by static seeding of CSCs for 2 days on each side. (ii) The valve structure went through rotary seeding of pVICs followed by static seeding of CSCs on both sides. (iii) The scaffolds were coated with 0.2 mg/ml fibronectin coating prior to the static seeding of pVICs followed by the seeding of CSCs. (iv) The scaffolds were coated with 0.2 mg/ml fibronectin coating followed by the rotary seeding of pVICs and static seeding of CSCs. In the rotary seeding, the density of pVICs was approximately 6000 cells/mm^3^ where the scaffolds were placed in a 50 ml Tube Spin® Bioreactor (TPP) with 25 ml of cell suspension in the complete medium. The scaffolds were rotated at 10 rpm for 15 days. For seeding of CSCs, the CSC growth medium was used along with a density of 6000 cells/mm^3^ per scaffold.

### Performance study of the developed Engineered Heart Valve structure

#### Pulse duplicator system

A pulse duplicator system (Aptus Bioreractors, Clemson SC USA) was used to expose heart valves to clinically relevant hemodynamic environment. PCL, 30:70 PCL: PLLA and commercial (Edwards 2800) valves were inserted to the valve housing of the system. The bioreactor platform was equipped with a holder system that enabled testing different scaffolds as heart valve leaflets. The holder was built out of transparent PDMS to enhance optical visibility. Geometry of the sinuses and leaflets could be freely chosen/designed to optimize the engineered heart valve. We compared the performance of PCL valve, PCL: PLLA valve, and commercially available prosthetic valve (Edwards 2800 valve). Systolic and diastolic pressures were generated by the bottom membrane of the bioreactor. The heart rate was selected 70 bpm, and systolic pressure was selected 20 mmHg in the control panel for the Aptus Pulse Duplicator System for all the tested valves. The system enables simultaneous monitoring of pressure value upstream of the valve (upper pressure) and downstream of the valve (lower pressure) as well as the flow rate. The pressure difference across the valves was calculated to define their function (Fig. 1E).

#### Doppler echocardiography of the developed engineered heart valve

Vivid-q ultrasonic medical imaging system (GE Healthcare) was integrated with the pulsed duplicator system for functional analysis of tested valves. The imaging system was used to visualize leaflet movements of the tested valves using the B-mode, and to measure the flow velocities through the valves at each pulse using the Doppler mode. Flow velocities indicate fluid shear stress levels on valve leaflets. Maximum and average values of both positive and negative velocities were calculated to characterize valve function. Positive velocity values indicate shear stress levels whereas negative velocity values indicate amount of regurgitation. Valve orifice size indicates the opening characteristics of the leaflets (Fig. 1E).

## Conclusions

In the current study, an engineered tri-leaflet heart valve structure is developed using electrospun PCL and PLLA scaffolds, and a set of *in vitro* testing protocol has been developed and demonstrated for *in vitro* performance study of tissue engineered heart valves. The *in vitro* testing protocol aims at understanding the microstructure, mechanical and biological properties of the scaffolds, and the hemodynamics of the developed engineered valves. The current study showed that PCL has higher stiffness and better mechanical properties compared to PLLA. However, the *in vitro* cell culture studies revealed that cells adhere preferentially on PLLA rather than on PCL. Thus, PCL can be used as stiffening and strengthening agent to improve the mechanical properties of scaffolds while PLLA can be used to improve their biological properties. Therefore, in the case of blends and mixtures of PCL and PLLA, the proliferation was improved with the increase in the percentage of PLLA. An evident boost in proliferation was noticed for 30:70 PCL: PLLA blend composition. Briefly, the blend or the mixture of PCL and PLLA retains the mechanical properties of PCL and the biological properties of PLLA. Therefore, the combination of the stiff PCL and the more compliant and cell friendly PLLA resulted in a scaffold with strength, pliability and biological properties suitable for applications in heart valve tissue engineering. The tri-leaflet heart valve structure engineered by incorporating a specially crafted PCL-PLLA scaffold onto a 3D printed stent were durable, rigid and sturdy, proving our simple technique to be very effective.

The three-dimensional cell seeding using the rotary dynamic seeding technique resulted in better penetration of valvular interstitial cells inside the pores of the scaffolds across its thickness resulting in improved cell colonization. The static surface seeding of cardiac stem cells on both sides helped in formation of a monolayer of CSCs, which lead the stem cell to colonize between the pores and to adhere functionally to the valve structure. Also, we were able to develop a flow loop pulsed duplicator system in combination with an ultrasound based experimental flow system enabling us to evaluate the hemodynamic performance of the engineered tri-leaflet heart valve *in vitro* in a biomimetic cardiac environment.

The current study has a number of limitations. For example, the Aptus pulsed duplicator system used in this study was only able to generate physiologic pressures for pulmonary circulation (~20 mmHg). A system capable of offering a range of pressure, or a higher pressure such as up to the aortic pressure (~90 mmHg) will be much more useful for better characterization of engineered valves. Such a system is currently under development and will be employed in future studies. Another limitation is: all of the characterizations performed in this article were *in vitro* characterization under the lab settings, whereas the *in vivo* performance may or may not be the same as *in vitro* ones. Moreover, even though the initial mechanical properties of the structure are shown to be very suitable for transplantation in human body, the mechanical durability over the time as the biodegradation of the scaffold will take place and new extracellular matrices will be deposited may or may not be better or similar to the initial properties. Thus further investigations are necessary through longer-term experiments, under various physiological conditions, as well as under *in vivo* environment in animal bodies and under conditions where the effect of biodegradation and matrix deposition can be well understood.

## References

[1] Sanz-Garcia A (2015). Heart valve tissue engineering: how far is the bedside from the bench?. Expert reviews in molecular medicine.

[2] Hasan, A. *et al*. Injectable hydrogels for cardiac tissue repair after myocardial infarction. *Advanced Science* **2** (2015). 10.1002/advs.201500122 PMC5033116 27668147

[3] d’Arcy J, Prendergast B, Chambers J, Ray S, Bridgewater B (2011). Valvular heart disease: the next cardiac epidemic. Heart.

[4] Rahimtoola SH (2003). Choice of prosthetic heart valve for adult patients. Journal of the American College of Cardiology.

[5] van Geldorp MW (2009). Patient outcome after aortic valve replacement with a mechanical or biological prosthesis: weighing lifetime anticoagulant-related event risk against reoperation risk. The Journal of thoracic and cardiovascular surgery.

[6] Kulik A (2010). Postoperative lipid-lowering therapy and bioprosthesis structural valve deterioration: justification for a randomised trial?. European Journal of Cardio-Thoracic Surgery.

[7] Shinoka T (2015). Current Status of Cardiovascular Tis-sue Engineering. Int J Clin Ther Diagn. S.

[8] Hammermeister KE (1993). A comparison of outcomes in men 11 years after heart-valve replacement with a mechanical valve or bioprosthesis. New England Journal of Medicine.

[9] Puvimanasinghe J (2001). Prognosis after aortic valve replacement with a bioprosthesis predictions based on meta-analysis and microsimulation. Circulation.

[10] Tillquist MN, Maddox TM (2011). Cardiac crossroads: deciding between mechanical or bioprosthetic heart valve replacement. Patient Prefer Adherence.

[11] Pesce, M. & Santoro, R. In *Regenerative Medicine-from Protocol to Patient* 1–12 (Springer, 2016).

[12] Hasan A (2014). Electrospun scaffolds for tissue engineering of vascular grafts. Acta biomaterialia.

[13] Colazzo F (2011). Extracellular matrix production by adipose-derived stem cells: implications for heart valve tissue engineering. Biomaterials.

[14] Murali, D., Kshirsagar, K. G., Hasan, A. & Paul, A. Harnessing the Potential of Stem Cells from Different Sources for Tissue Engineering. *Tissue Engineering for Artificial Organs: Regenerative Medicine, Smart Diagnostics and Personalized Medicine*, 85–109 (2017).

[15] Alavi SH, Ruiz V, Krasieva T, Botvinick EL, Kheradvar A (2013). Characterizing the collagen fiber orientation in pericardial leaflets under mechanical loading conditions. Annals of biomedical engineering.

[16] Hasan A (2016). Engineered Biomaterials to Enhance Stem Cell‐Based Cardiac Tissue Engineering and Therapy. Macromolecular bioscience.

[17] Hasan A (2014). Microfluidic techniques for development of 3D vascularized tissue. Biomaterials.

[18] Annabi N (2013). Elastomeric recombinant protein-based biomaterials. Biochemical engineering journal.

[19] Suntornnond R, An J, Yeong WY, Chua CK (2015). Biodegradable polymeric films and membranes processing and forming for tissue engineering. Macromolecular Materials and Engineering.

[20] Hasan A (2014). Biomechanical properties of native and tissue engineered heart valve constructs. Journal of Biomechanics.

[21] Deeb, G., Hasan, A., Abiad, M., Alhadrami, H. A. & Mustafy, T. In *Advances in Biomedical Engineering (ICABME)*, *2015 International Conference on*. 226–229 (IEEE).

[22] Sacks MS, Yoganathan AP (2007). Heart valve function: a biomechanical perspective. Philosophical Transactions of the Royal Society of London B: Biological Sciences.

[23] Sant S, Iyer D, Gaharwar AK, Patel A, Khademhosseini A (2013). Effect of biodegradation and de novo matrix synthesis on the mechanical properties of valvular interstitial cell-seeded polyglycerol sebacate–polycaprolactone scaffolds. Acta biomaterialia.

[24] Goonoo N (2015). Poly (ester-ether) s: III. assessment of cell behaviour on nanofibrous scaffolds of PCL, PLLA and PDX blended with amorphous PMeDX. Journal of Materials Chemistry B.

[25] Hasan A (2016). Micro and nanotechnologies in heart valve tissue engineering. Biomaterials.

[26] Vrana, N. E. Cell and Material Interface: Advances in Tissue Engineering, Biosensor, Implant, and Imaging Technologies (CRC Press, 2015).

[27] Barthes, J. *et al*. Cell microenvironment engineering and monitoring for tissue engineering and regenerative medicine: the recent advances. *BioMed research international* **2014** (2014). 10.1155/2014/921905 PMC4124711 25143954

[28] Lieshout MV, Peters G, Rutten M, Baaijens F (2006). A knitted, fibrin-covered polycaprolactone scaffold for tissue engineering of the aortic valve. Tissue engineering.

[29] Ramakrishna S, Mayer J, Wintermantel E, Leong KW (2001). Biomedical applications of polymer-composite materials: a review. Composites science and technology.

[30] Yue K (2015). Synthesis, properties, and biomedical applications of gelatin methacryloyl (GelMA) hydrogels. Biomaterials.

[31] Masoumi N (2014). Tri-layered elastomeric scaffolds for engineering heart valve leaflets. Biomaterials.

[32] Hong Z, Reis RL, Mano JF (2009). Preparation and *in vitro* characterization of novel bioactive glass ceramic nanoparticles. Journal of biomedical materials research Part A.

[33] Patricio T, Gloria A, Bartolo P (2013). Mechanical and biological behaviour of PCL and PCL/PLA scaffolds for tissue engineering applications. Chem Eng Trans.

[34] Chen L (2013). Electrospun poly (l-lactide)/Poly (ε-caprolactone) blend nanofibrous scaffold: characterization and biocompatibility with human adipose-derived stem cells. Plos One.

[35] Johnson CM, Hanson MN, Helgeson SC (1987). Porcine cardiac valvular subendothelial cells in culture: cell isolation and growth characteristics. Journal of molecular and cellular cardiology.

[36] Forte G (2008). Criticality of the biological and physical stimuli array inducing resident cardiac stem cell determination. Stem Cells.

[37] Barile L (2007). Cardiac stem cells: isolation, expansion and experimental use for myocardial regeneration. Nature Reviews. Cardiology.

